# Molecular survey on vector-borne pathogens in clinically healthy stray cats in Zaragoza (Spain)

**DOI:** 10.1186/s13071-023-06046-y

**Published:** 2023-11-20

**Authors:** Sergio Villanueva-Saz, Marivi Martínez, Ard M. Nijhof, Bastian Gerst, Michaela Gentil, Elisabeth Müller, Antonio Fernández, Ana González, Mohamed Sh. Mohamud Yusuf, Grazia Greco, Maite Verde, Giovanni Sgroi, Delia Lacasta, Diana Marteles, Michele Trotta, Ingo Schäfer

**Affiliations:** 1 Immunology Laboratory, Zaragoza Veterinary Faculty, Zaragoza University, Miguel Servet 177, 50013 Zaragoza, Spain; 2 Animal Pathology Department, Zaragoza Veterinary Faculty, Zaragoza University, Miguel Servet 177, 50013 Zaragoza, Spain; 3 Instituto Agroalimentario de Aragón‑IA2 (Universidad de Saragossa‑CITA), Zaragoza University, Miguel Servet 177, 50013 Zaragoza, Spain; 4 Institute for Parasitology and Tropical Veterinary Medicine, Freie Universität Berlin, Robert-Von-Ostertag-Straße 7, 14163 Berlin, Germany; 5 Veterinary Centre for Resistance Research, Freie Universität Berlin, 14163 Berlin, Germany; 6 LABOKLIN GmbH and Co. KG, Steubenstraße 4, 97688 Bad Kissingen, Germany; 7 Hospital Veterinario Universidad de Zaragoza, Zaragoza University, Miguel Servet 177, 50013 Zaragoza, Spain; 8 Department of Veterinary Medicine, University of Bari “Aldo Moro”, 70010 Valenzano, Metropolitan City of Bari Italy; 9 Department of Animal Health, Experimental Zooprophylatic Institute of Southern Italy, Portici, 80055 Naples, Italy

**Keywords:** Arthropod-transmitted infections, Feline vector-borne infections, Laboratory diagnostics, PCR, Tick-transmitted infections

## Abstract

**Background:**

In Europe, feline vector-borne infections are gaining importance because of the changing climate, expanding habitats of potential vectors and expanding pathogen reservoirs. The main objective of this study was to assess the prevalence of vector-borne pathogens (VBPs) in stray cats in Zaragoza, Spain, and to investigate potential risk factors for infection, including feline leukaemia virus (FeLV) and feline immunodeficiency virus (FIV).

**Methods:**

Blood samples from stray cats presented to the veterinary faculty in Zaragoza between February 2020 and 2022 were tested by polymerase chain reaction (PCR) for the presence of *Anaplasma phagocytophilum*, *Anaplasma platys*, *Bartonella henselae*, *Ehrlichia canis*, *Rickettsia* spp., haemotropic *Mycoplasma* spp., *Hepatozoon* spp., *Leishmania infantum*, piroplasms and microfilariae at the LABOKLIN laboratory. The cats were also tested for FeLV and FIV by PCR.

**Results:**

Nearly half of the cats (158/332, 47.6%) were positive for at least one VBP. *Hepatozoon* spp. were detected in 25.6%, haemotropic *Mycoplasma* spp. in 22.9%, *B. henselae* in 9.3% and *L. infantum* in 2.1% of the cats. Male sex had a statistically significant association with test results for haemotropic *Mycoplasma* spp. (odds ratio 1.38 [1.21;1.57]); regionality with *Hepatozoon* spp., *B. henseale* and FIV; and seasonality with *Hepatozoon* spp., haemotropic *Mycoplasma* spp., *L. infantum* and FeLV (*P* ≤ 0.05 each). A strong positive correlation was reported for the amount of rainfall and the number of cats that tested positive for *Hepatozoon* spp. (*ρ* = 753, *P* = 0.05). None of the cats tested positive for *A. phagocytophilum*, *A. platys*, *E. canis*, *Rickettsia* spp., piroplasms, or microfilariae. Co-infections with multiple VBPs were detected in 56 out of 332 cats (16.9%). Thirty-one of the 332 cats included in the study (9.3%) tested positive for FeLV (6.9%) and for FIV (3.6%). In 20/31 cats (64.5%) that tested positive for FeLV/FIV, coinfections with VBP were detected (*P* = 0.048, OR 2.15 [0.99; 4.64]).

**Conclusions:**

VBPs were frequently detected in stray cats in Zaragoza. In particular, regionality and seasonality had a statistically significant association with PCR results for most VBPs included in the study.

**Graphical Abstract:**

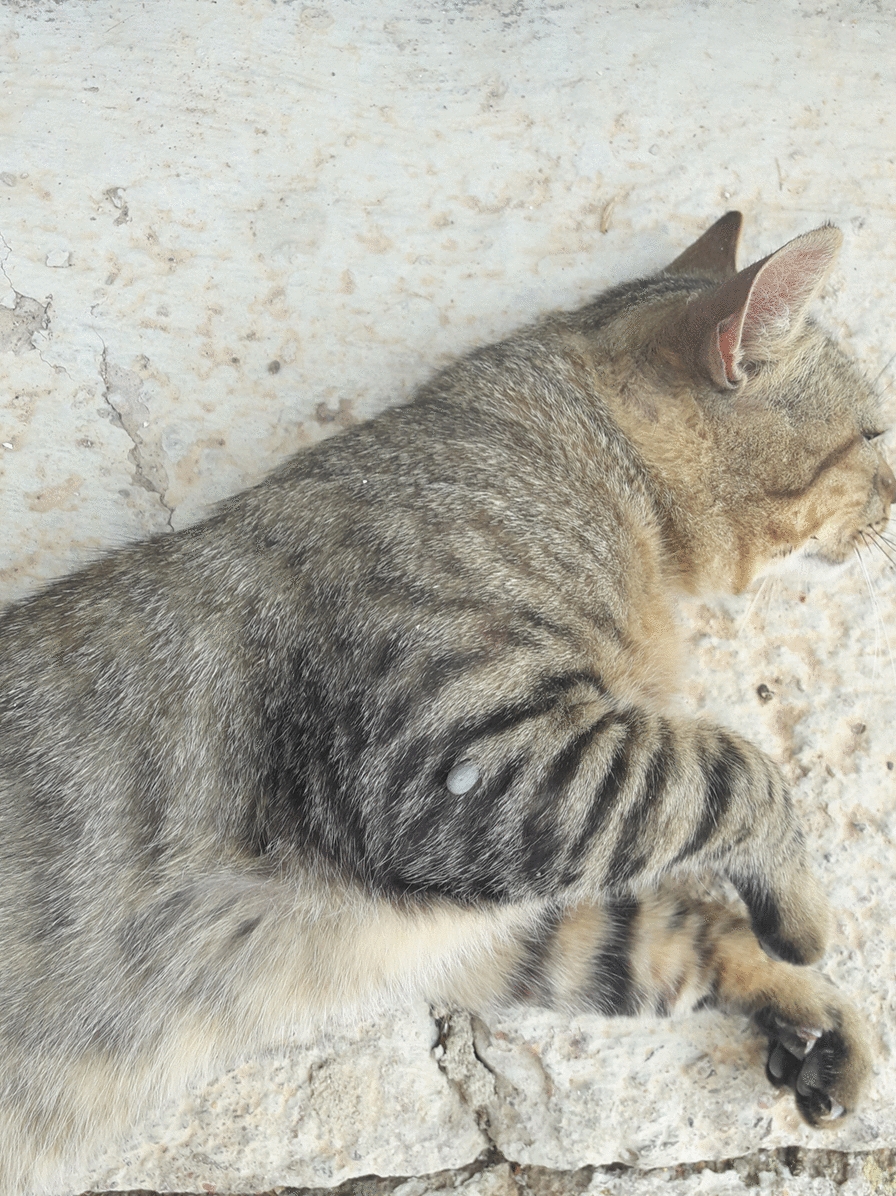

**Supplementary Information:**

The online version contains supplementary material available at 10.1186/s13071-023-06046-y.

## Highlights

- Nearly half of the cats tested positive for at least one vector-borne pathogen by PCR
- *Hepatozoon felis*, haemotropic *Mycoplasma*, *Bartonella* and *Leishmania* were detected
- Stray cats should be monitored to prevent and manage potential zoonotic diseases

**Supplementary Information:**

The online version contains supplementary material available at 10.1186/s13071-023-06046-y.

## Introduction

The importance of feline vector-borne pathogens (VBPs), transmitted by blood-feeding arthropods, is increasingly being recognized in Europe. Cats are exposed to arthropods, especially when living outdoors or as stray cats without ectoparasite prophylaxis and veterinary care [1–3]. Ectoparasites can transmit various parasitic, bacterial, or viral pathogens to animal hosts such as cats. Cats with immunosuppression as a result of feline leukaemia virus (FeLV) and feline immunodeficiency virus (FIV) infections are predisposed to vector-borne infections including leishmaniosis [4], hepatozoonosis [5–7] and cytauxzoonosis [8]. Screening for VBPs may be pivotal for animal and environmental risk assessment.

The distribution of feline VBPs is mainly dependent on the habitats of the transmitting vectors [2]. Consequently, *Anaplasma phagocytophilum* infections have most often been recognized in northern, central, or eastern Europe [9]. The occurrence of *Leishmania infantum* infections has been linked to habitats of phlebotomine sand flies in the Mediterranean and Southeastern Europe [9]. Infections with *Hepatozoon felis* and less frequently with *H. canis* and *H. silvestris* were detected in the Mediterranean and Southeastern Europe [10–14]. Feline infections with *Dirofilaria* *immitis* are mainly reported within the Mediterranean and Southeastern Europe [15, 16]. Finally, infections with *Dirofilaria repens* are rare in cats [17]. Other documented VBPs affecting cats in Europe include helminths (e.g. *Thelazia callipaeda*, *Dipylidium caninum*), bacteria (e.g. *Borrelia burgdorferi* sensu lato, *Bartonella* spp., *Francisella tularensis*), protozoa (e.g. *Babesia* spp., *Theileria* spp., *Cytauxzoon* sp.) and viruses (Flaviviridae) [2]. For many of these feline VBPs, infections in humans have been reported as well. This, for example, includes *Bartonella* *henselae* and *B. clarridgeiae* as the causative agents of cat scratch disease in humans [18], *Leishmania infantum* causing human leishmaniosis [19], *A. phagocytophilum* causing human granulocytic anaplasmosis [20], *D. repens* causing subcutaneous dirofilariosis [21] and *D. immitis* causing heartworm infections [21]. Haemotropic *Mycoplasma* spp. are bacteria that are classified within the *Mycoplasma* genus but cannot be cultivated [22]. *Mycoplasma haemofelis*, *Candidatus Mycoplasma haemominutum* and *Candidatus Mycoplasma turicensis* are the most commonly detected species in cats [23].

The aims of this study were to investigate the prevalence of several VBPs in stray cats in the city of Zaragoza, Spain, and to identify potential risk factors for infection.

## Materials and methods

### Study areas, cat population and sampling

The study was carried out in the city of Zaragoza (41°38′58.8948ʺN, 0°53′15.7632ʺW, the Aragon region of Spain). The study population included stray European shorthair cats captured in different areas of Zaragoza from February 2020 to October 2022 as part of a trap, neuter and release sterilization programme that ran locally to control stray populations. Captured stray cats were anaesthetized with a combination of dexmedetomidine (Dexdomitor®; 15 µg/kg, subcutaneous injection), ketamine (Anaestamine®; 5 mg/kg, subcutaneous injection) and methadone (Semfortan®; 0.3 mg/kg, subcutaneous injection). Data on the breed, sex, blood collection date and colony of origin (postal code) of each cat were recorded. A complete physical examination was carried out before sampling. Only cats older than 1 year and classified as apparently healthy based on the general examination were included. Prior to blood collection, the fur of the cats was trimmed around the jugular region. Sampling consisted of collecting 1 ml of blood aseptically by jugular venepuncture, with the collected volume placed in a tube containing ethylenediaminetetraacetic acid (EDTA) anticoagulant for PCR analysis. Blood was stored at − 20 °C until processing.

This survey was included under Project Licence PI75/20 approved by the Ethics Committee for Animal Experiments for the University of Zaragoza. The care and use of animals were performed according to the Spanish Policy for Animal Protection RD 53/2013, which meets the European Union Directive 2010/63 on the protection of animals used for experimental and other scientific purposes.

### Molecular analysis

PCR testing of EDTA-blood was performed to detect *A. phagocytophilum*, *A. platys*, *B. henselae*, *E. canis*, *Rickettsia* spp., haemotropic *Mycoplasma* spp., *Hepatozoon* spp., *L. infantum*, piroplasms and microfilariae in the LABOKLIN laboratory (Bad Kissingen, Germany). Additionally, PCR testing was performed for FeLV and FIV. The molecular methods applied for investigation with descriptions of the gene targets at the LABOKLIN laboratory are summarized in Table 1. Furthermore, additional PCR protocols were used for sequence analysis of *Bartonella* spp. and *Hepatozoon* spp. (Table 1).

**Table 1:** Methodology of PCR testing applied for the detection of vector-borne pathogens performed in the present study

Vector-borne pathogen	Molecular method	Gene target	Primer sequence (5’-3 ‘)
*Anaplasma phagocytophilum*	TaqMan® real-time PCR	Heat shock protein 60	F: CTCTGAGCACGCTTGTACT R: GCCTTTACAGCAGCAACTTGAAG
*Anaplasma platys*	TaqMan® real-time PCR	groEL	F: CCGATCCTTGAAAACGTTGCT R: TCCTTCTACATCCTCAGCGATGAT
*Ehrlichia canis*	TaqMan® real-time PCR	Disulphide-oxidoreductase	F: CCCTCAAAAAGTGATAGATCTGCTCA R: TCCAATTCAGATTTGTATTTTTTTATGTTTTGACTCA
*Rickettsia* spp.	TaqMan® real-time PCR	23S rRNA	F: AGCTTGCTTTTGGATCATTTGG R: TTCCTTGCCTTTTCATACATCTAGT
*Mycoplasma haemofelis*	TaqMan® real-time PCR	16S rDNA	F: GTGCTACAATGGCGAACACA R: TCCTATCCGAACTGAGACGAA
*Mycoplasma haemominutum*	TaqMan® real-time PCR	16S rDNA	F: TGATCTATTGTKAAAGGCACTTGCT R: TTAGCCTCYGGTGTTCCTCAA
*Mycoplasma turicensis*	TaqMan® real-time PCR	16S rDNA	F: AGAGGCGAAGGCGAAAACT R: CTACAACGCCGAAACACAAA
*Bartonella henselae* (for detection)	TaqMan® real-time PCR	Alr-gcvP IGS	F: GAGGGAAATGACTCTCTCAGTAAAA R: TGAACAGGATGTGGAAGAAGG
*Bartonella spp.* (for typing)	cPCR	16S—23S ITS	F: CTTCAGATGATGATCCCAAGCCTTYTGGCG R: GAACCGACGACCCCCTGCTTGCAAAGCA
*Bartonella henselae* (for typing, I or II)	cPCR	16S rRNA	Bh_F: AGAGTTTGATCCTGG(CT)TCAG BhI_R: CCGATAAATCTTTCTCCCTAA BhII_R: CCGATAAATCTTTCTCCAAAT
*Leishmania infantum*	TaqMan® real-time PCR	Kinetoplast minicircle DNA	F: AACTTTTCTGGTCCTCCGGGTAG R: ACCCCCAGTTTCCCGCC
*Hepatozoon* spp. (for detection)	TaqMan® real-time PCR	18S rRNA	F: AACACGGGAAAACTCACCAG R: CCTCAAACTTCCTCGCGTTA
*Hepatozoon* spp. (for typing)	cPCR and sequencing	18S rRNA	F: CAGTAAAACTGCAAATGGCTCAT R: CCAATAATGTAGAACCAAAATCCT
Piroplasms	Conventional PCR	Small subunit ribosomal	F: AATACCCAATCCTGACACAGGG R: TTAAATACGAATGCCCCCAAC
Microfilariae	TaqMan® real-time PCR	5.8S rDNA	F: AGTGCGAATTGCAGACGC R: ATTGACCCTCAACCAGACG
FeLV	TaqMan® real-time PCR	U3 long terminal repeat	F: TCAAGTATGTTCCCATGAGATACAA R: GAAGGTCGAACTCTGGTCAACT
FIV	TaqMan® real-time PCR	Gag	F: GGCATATCCTATTCAAACAG R: AAGAGTTGCATTTTATATCC

### *Bartonella* and *Hepatozoon* species identification and sequence analysis

For *Bartonella* sp. identification, samples that tested positive with *Bartonella* sp. qPCR were submitted to a conventional PCR (cPCR) assay amplifying an ~ 603 bp fragment of the 16S-23S intergenic transcribed spacer (ITS) region (Table 1) [24]. The cPCR cycling conditions consisted of an initial denaturation step at 94 °C for 2 min, followed by 40 cycles of 94 °C for 15 s, 60 °C for 15 s and 72 °C for 45 s. The ITS cPCR products were sequenced for further identification and characterization. Furthermore, the partial 16S rRNA fragment from all ITS cPCR positive samples was amplified for genotype characterization by using the type-specific primers Bh1_R (5′- CCGATAAATCTTTCTCCCTAA -3′) and Bh2_R (5′- CCGATAAATCTTTCTCCAAAT -3′) in combination with the broad-host-range primer 16SF [5′-AGAGTTTGATCCTGG(CT)TCAG-3′] as described previously (51) (Table 1). The cPCR cycling conditions consisted of an initial denaturation step at 94 °C for 2 min, followed by 40 cycles of 94 °C for 15 s, 57 °C for 15 s and 72 °C for 15 s. All cPCR assays were performed in a final volume of 25 µl that contained 2 µl of DNA extract, 2.5 µl of 10 × TaKaRa LA Taq buffer, 0.25 µl of TaKaRa LA Taq enzyme (Takara Bio Europe S.A.S.Saint-Germain-en-Laye, France), 2.5 µl of MgCl2 (25 mM), 1 µl each of forward and reverse primers (50 µM), 4 µl of dNTPs and PCR grade water up to 25 µl. For species and genotype characterization, the obtained ITS cPCR products were sequenced in both directions using BigDye 3.1 Ready Reaction Mix (Applied Biosystems) according to the manufacturer’s instructions.

For *Hepatozoon* species identification, a 779-bp fragment of the 18S rRNA gene was amplified using the primers Hep18S-F74 (5’- CAGTAAAACTGCAAATGGCTCAT-3’) and Hep18S-R853 (5’-CCAATAATGTAGAACCAAAATCCT-3’). The 25 µl PCR mixture contained the following: 15.25 µl PCR-grade water, 5 µl 5 × reaction buffer (Biozym, Oldendorf, Germany), 1 µl of each primer (10 pmol), 0.25 µl of Biozym HS Taq DNA Polymerase and 2.5 µl template DNA. The cPCR cycling conditions consisted of an initial denaturation step at 98 °C for 2 min followed by 40 cycles of 98 °C for 15 s, 55.5 °C for 15 s and 72 °C for 15 s. *Hepatozoon* 18S rRNA amplicons were Sanger sequenced by LGC Genomics, Berlin, Germany.

The genomic sequences obtained in this study were edited using Geneious 10.1.3 2020 version (Biomatters Ltd., Auckland, New Zealand) and submitted to a BLAST search (BLASTn; https://blast.ncbi.nlm.nih.gov/Blast.cgi?PROGRAM=blastn&PAGE_TYPE=BlastSearch&LINK_LOC=blasthome) using default values to find homologous hits. For the phylogenetic analyses, sequences were aligned using the L-INS-I algorithm of the Geneious MAFFT plugin with default settings. A maximum likelihood (ML) phylogenetic tree with 1000 bootstrap resamplings using the GTR-GAMMA nucleotide model was performed using the Geneious RAxML plugin.

### Statistical analysis

Statistical analysis was carried out using SPSS for Windows (version 28.0; IBM). *P* ≤ 0.05 was regarded as statistically significant. The dataset was tested for adherence to a normal distribution in cats that tested positive for VBPs by Shapiro-Wilk testing. For normally distributed data, the chi-square test and Fisher’s exact test were used to calculate statistical significance. For data on seasonality, which were not normally distributed, Kruskal-Wallis testing was used to calculate statistical significance. The different seasons were defined as follows: spring from 21 March to 20 June, summer from 21 June to 22 September, autumn from 23 September to 31 December and winter from 1 January to 20 March. Binomial logistic regression was performed to determine the effect of sex, regional distribution and seasonality on the PCR test results.

Spearman’s rank correlation coefficient (*ρ*) was calculated to correlate the percentage of cats testing positive for the individual VBPs with the amounts of rainfall and average temperatures in the individual months of the study (https://www.aemet.es/es/serviciosclimaticos/datosclimatologicos). *ρ* < 0.2 was classified as a very low correlation, *ρ* = 0.2–0.5 as a low correlation, *ρ* > 0.5–0.7 as a medium correlation, *ρ* > 0.7–0.9 as a strong correlation and *ρ* > 0.9 as a very strong correlation.

## Results

Out of the 332 cats included in this study, 158 (47.6%) tested positive for at least one VBP (Table 2). *Hepatozoon* spp. (85 out of 332 cats, 25.6%), haemotropic *Mycoplasma* spp. (76 out of 332 cats, 22.9%), *B. henselae* (31 out of 332 cats, 9.3%) and *L. infantum* (7 out of 332 cats, 2.1%) were detected. None of the cats tested positive for *A. phagocytophilum*, *A. platys*, *E. canis* or *Rickettsia* spp. Twenty-three of 332 cats (6.9%) tested PCR positive for FeLV, and 12 of 332 cats (3.6%) tested PCR positive for FIV. In 21 of the 31 cats that tested positive for FeLV and/or FIV, coinfections with VBPs were detected (*P* = 0.048, OR = 2.15 [95% CI 0.99–4.64]).

**Table 2:** Proportions of the molecularly detected pathogens in stray cats from Zaragoza, Spain

Pathogen	*n* tested positive/*N* total	Percentage (%)
Bacterial pathogens		
Haemotropic *Mycoplasma* spp.	76/332	22.9
*Candidatus* M. hemominutum	61/332	18.4
*M. hemofelis*	35/332	10.5
*Candidatus* M. turicensis	11/332	3.3
*Bartonella* spp.	31/332	9.3
*B. henselae* type I	1/332	0.30
*B. henselae* type II	28/332	8.73
*Bartonella* spp.	2/332	0.6
*Anaplasma phagocytophilum*	0/332	0
*Anaplasma platys*	0/332	0
*Ehrlichia canis*	0/332	0
*Rickettsia* spp.	0/332	0
Protozoal pathogens		
*Hepatozoon* spp.	85/332	25.6
*Leishmania infantum*	7/332	2.1
Piroplasma	0/332	0
Helminths		
Microfilariae	0/332	0
Viral pathogens		
Feline leukaemia virus (FeLV)	23/332	6.9
Feline immunodeficiency virus (FIV)	12/332	3.6

In 56/158 cats (35.4%) that tested positive, co-infections with more than one vector-borne pathogen were recognized. Most often, haemotropic *Mycoplasma* spp. (46 out of 56 cats, 82.1%) were involved, followed by *Hepatozoon* spp. (29 out of 56 cats, 51.8%), *B. henselae* (17 out of 56 cats, 30.4%) and *L. infantum* (5 out of 56 cats, 8.9%). In 43 out of 56 cats (76.8%), co-infections with two pathogens were recognized; in 12 cats (21.4%), co-infections with three pathogens; in one cat (1.8%), co-infections were with four pathogens.

Of 31 samples that yielded amplicons of the expected size when using cPCR targeting the 16S-23S ITS region of *Bartonella* spp., 28 (96.6%) were characterized as 16S *B. henselae* type II and one as *B. henselae* type I based on the 16S rRNA cPCR assay (Table 2). The identity of the two remaining samples (cats #48 and #122) could not be determined because of the low quantity of the available DNA. Of the ITS-positive samples, 11/29 (37.5%) were successfully sequenced. Ten of the sequences were identical, so only two sequences were deposited in GenBank® under accession numbers: OQ918678 and OQ918679. According to the sequence data, *B. henselae* was the only species present with two different ITS sequence types detected that matched with the partial ITS sequences of the reference strains Houston I (BAO16RB) (catalogue #284–OQ918679) and Marseille II (CP072904) (catalogue #196–OQ918678), as stated in Additional file 1: Fig. S1.

A partial fragment of the 18S rRNA gene from *Hepatozoon* species was successfully amplified from 70 samples that tested positive in TaqMan real-time PCR for *Hepatozoon* spp. Nineteen *H. felis* sequence variants (variants A–S, OR263276-OR263294) were detected that showed 99.1–100% identity with the 18S rRNA gene of a Spanish *H. felis* isolate (AY620232). In one cat (catalogue #98), an *H. canis* sequence (OR263295) that was 100% identical to another *H. canis* isolate from Spain (AY150067) was detected (Additional file 1: Fig. S2). Considering regional distribution, sequence variants were different between areas: Central (variants A, D, G, O, Q and S), North (variants A, E, M and Q), South (variants A, E, F, I, O, P and Q), East (variants A, H, Q, R and S) and West (variants A, J, K and S).

Of 332 cats, 179 (53.9%) were female, and 153 (46.1%) were male. A statistically significant impact of sex on the test results was detected only for haemotropic *Mycoplasma* spp. (21/179 female [11.7%] and 55/153 male cats [35.9%] tested positive, *P* < 0.001, OR = 1.38; in 21 of the 31 cats testing positive for FeLV and/or FIV, coinfections with VBPs were detected (*P* = 0.048, OR = 2.15 [95% CI 1.21 -1.57]). There was no statistically significant impact of sex on *Hepatozoon* spp. (47/179 female [26.3%] and 38/153 male cats [24.8%] tested positive, *P* = 0.802), *L. infantum* (2/179 female [1.1%] and 5/153 male cats [3.3%] tested positive, *P* = 0.255), *B. henselae* (16/179 female [8.9%] and 15/153 male cats [9.8%] tested positive, *P* = 0.851), FeLV (8/179 female [4.5%] and 15/153 male cats [9.8%] tested positive,* P* = 0.081) or FIV (3/179 female [1.7%] and 9/153 male cats [5.9%] tested positive, *P* = 0.073).

The postal code of the area in which the cats were caught in Zaragoza was known for 322/332 cats (97.0%). There were statistically significant differences between regions and PCR positivity for *Hepatozoon* spp. (*χ*^*2*^ = 17.396, *df* = 4, *P* = 0.002), *B. henselae* (*χ*^*2*^ = 14.544, *df* = 4, *P* = 0.006) and FIV (*χ*^*2*^ = 11.961, *df* = 4, *P* = 0.018), which could not be demonstrated in haemotropic *Mycoplasma* spp. (*χ*^*2*^ = 4.395, *df* = 4, *P* = 0.355),* L. infantum* (*χ*^*2*^ = 7.523, *df* = 4, *P* = 0.111) and FeLV (*χ*^*2*^ = 3.060, *df* = 4, *P* = 0.548) (Table 3).

**Table 3:** Regional distribution of positive molecular test results for selected vector-borne pathogens and FeLV/FIV in stray cats in Zaragoza, Spain, according to postal codes (*n* tested positive/*N* total [%])

Pathogen	Central	North	South	East	West	Total	*P*
*Hepatozoon* spp.	27/131 (20.6%)	9/60 (15%)	12/25 (48%)	22/56 (39.3%)	15/50 (30%)	85/322 (26.4%)	0.002
Haemotropic* Mycoplasma spp.*	24/131 (18.3%)	17/60 (28.3%)	8/25 (32%)	12/56 (21.4%)	9/50 (18%)	70/322 (21.7%)	0.355
*Bartonella henseale*	5/131 (3.8%)	9/60 (15%)	0/25 (0%)	6/56 (10.7%)	9/50 (18%)	29/322 (9.0%)	0.006
*Leishmania infantum*	1/131 (0.8%)	4/60 (6.7%)	0/25 (0%)	1/56 (1.8%)	1/50 (2.0%)	7/322 (2.2%)	0.111
FeLV	13/131 (9.9%)	3/60 (5%)	1/25 (4%)	4/56 (7.1%)	2/50 (4%)	23/322 (7.1%)	0.548
FIV	8/131 (6.1%)	0/60 (0%)	0/25 (0%)	0/56 (0%)	0/50 (0%)	8/322 (2.5%)	0.018
Total	131/322 (40.7%)	60/322 (18.6%)	25/322 (7.8%)	56/322 (17.4%)	50/322 (15.5%)	322/322 (100%)	**–**

Central: 50,001, 50,003, 50,004, 50,005, 50,009, 50,010, 50,012, 50,014, 50,017, 50,018, 50,190, 50,191, 50,720

North: 50,015, 50,019, 50,020, 50,193, 50,820

South: 50,007, 50,021

East: 50,002, 50,013, 50,016, 50,057, 50,194

West: 50,011, 50,197, 50,620

*FeLV* Feline leukaemia virus, *FIV* feline immunodeficiency virus

Positive test results were not normally distributed for seasonality in cats testing positive for VBPs, FeLV and/or FIV (*P* < 0.001 each). Significant differences between seasons and PCR test results were observed for *Hepatozoon* spp. (*P* < 0.001), haemotropic *Mycoplasma* spp. (*P* = 0.048), *L. infantum* (*P* = 0.007) and FeLV (*P* = 0.045) when using the Kruskal-Wallis test but not for *B. henselae* (*P* = 0.111) and FIV (*P* = 0.674) (Table 4). Statistically significant differences in PCR results were observed when autumn and winter were compared for *Hepatozoon* spp. (*P* = 0.030, *r* = 0.2), haemotropic *Mycoplasma* spp. (*P* = 0.049, *r* = 0.2), *L. infantum* (*P* = 0.019, *r* = 0.3) and FeLV (*P* = 0.036, *r* = 0.2). Additionally, this was observed for spring and winter in *Hepatozoon* spp. (*P* < 0.001, *r* = 0.4) and for summer and winter in *L. infantum* (*P* = 0.010, *r* = 0.3) with weak to moderate effect sizes.

**Table 4:** Seasonal distribution of positive molecular test results for selected vector-borne pathogens and FeLV/FIV in stray cats in Zaragoza, Spain (*n* tested positive/*N* total [%])

Pathogen	Spring	Summer	Autumn	Winter	Total	*P*^1^
*Hepatozoon* spp.	28/71 (39.4)	20/83 (24.1)	31/111 (27.9)	6/67 (9.0)	85/332 (25.6)	< 0.001
Haemotropic* Mycoplasma spp.*	18/71 (25.4)	16/83 (19.3)	19/111 (17.1)	23/67 (34.3)	76/332 (22.9)	0.048
*Bartonella henselae*	6/71 (8.5)	3/83 (3.6)	12/111 (10.8)	10/67 (14.9)	31/332 (9.3)	0.111
*Leishmania infantum*	1/71 (1.4)	0/83 (0)	1/111 (0.9)	5/67 (7.5)	7/332 (2.1)	0.007
Total	39/71 (54.9)	33/83 (39.8)	51/111 (45.9)	35/67 (52.2)	158/332 (47.6)	0.233
FeLV	4/71 (5.6)	7/83 (8.4)	12/111 (10.8)	0/67 (0)	23/332 (6.9)	0.045
FIV	2/71 (2.8)	2/83 (2.4)	4/111 (3.6)	4/67 (6.0)	12/332 (3.6)	0.674

Spring: 21 March–20 June; summer: 21 June–22 September; autumn: 23 September–31 December; winter = 1 January–20 March

*FeLV* feline leukaemia virus; *FIV* feline immunodeficiency virus

^1^Kruskal-Wallis test

In binominal logistic regression analysis including 322 cats, seasonality with spring/autumn compared to summer/winter (*B* = 0.823, Wald = 8.867, *P* = 0.003, OR = 2.28 [95% CI 1.33–3.92]) and regionality with southern parts of Zaragoza compared to all other areas (*B* = 1.195, Wald = 7.48, *P* = 0.006, OR = 3.30 [95% CI 1.40–7.78]) contributed significantly to positive test results for *Hepatozoon* spp. but not male sex compared to female sex (*P* = 0.945). The binomial logistic regression model was statistically significant (*χ*^2^(3) = 15.233, *P* = 0.002). Goodness of fit was assessed using the Hosmer-Lemeshow test, indicating a good model fit (*χ*^2^(3) = 0.731, *P* > 0.05). Correlations between predictor variables were low (*r* < 0.70), indicating that multicollinearity was not a confounding factor in the analysis.

For haemotropic *Mycoplasma* spp., the binomial logistic regression model was statistically significant (*χ*^2^(3) = 30.557, *P* < 0.001). Goodness of fit was assessed using the Hosmer-Lemeshow test, indicating a good model fit (*χ*^2^(3) = 1.200, *P* > 0.05). Correlations between predictor variables were low (*r* < 0.70), indicating that multicollinearity was not a confounding factor in the analysis. Male sex had a statistically significant impact on the PCR results (*B* = 1.477, Wald = 23.747, *P* < 0.001, OR = 4.38 [95% CI 2.42–7.94]). Regionality with southern parts compared to all others (*P* = 0.104) and seasonality with winter compared to all other seasons (*P* = 0.246) did not show statistical significance.

For *B. henseale*, the binomial logistic regression model did not show any statistically significant impact of regionality (southern areas compared to all others, *P* = 0.989), seasonality (winter compared to all other seasons, *P* = 0.146) or male sex (*P* = 0.624) on the results of PCR-testing.

A strong positive correlation was reported for the amounts of rainfall and the percentages of cats testing positive for *Hepatozoon* spp. (*ρ* = 753, *P* = 0.05, Table 5). For haemotropic *Mycoplasma* spp. and *L. infantum*, no statistically significant correlations were detected with the amounts of rainfall (Additional file 1: Fig. S3) and the average temperature (Additional file 1: Fig. S4) in the individual months of the study (*P* > 0.05 each) (Table 5).

**Table 5:** Correlation between percentages of cats tested positive for haemotropic *Mycoplasma* spp., *Hepatozoon* spp. and *Leishmania infantum* in the individual months of the study with amounts of rainfall (mm) and temperature (°C) using Spearman’s rank correlation coefficient

		*Mycoplasma* spp.	*Hepatozoon* spp.	*Leishmania* spp.	Rainfall (mm)	Temperature (°C)
Haemotropic *Mycoplasma* spp.	CC	1.000	-0.512	0.484	-0.081	0.385
	Sig	–	0.089	0.111	0.803	0.216
	N	12	12	12	12	12
*Hepatozoon* spp.	CC	− 0.512	1.000	− 0.465	0.753	0.238
	Sig	0.089	−	0.128	0.005	0.456
	N	12	12	12	12	12
*Leishmania infantum*	CC	0.484	− 0.465	1.000	− 0.078	− 0.382
	Sig	0.111	0.128	–	0.810	0.220
	N	12	12	12	12	12
Rainfall (mm)	CC	− 0.081	0.753	− 0.078	1.000	− 0.154
	Sig	0.803	0.005	0.810	–	0.633
	N	12	12	12	12	12
Temperature (°C)	CC	− 0.385	0.238	− 0.382	− 0.154	1.000
	Sig	0.261	0.456	0.220	0.633	–
	N	12	12	12	12	12

## Discussion

Almost half of the cats in our study (47.6%) tested positive for at least one VBP by molecular analysis. This is highly indicative of infection, in contrast to positive serological antibody detection demonstrating pathogen contact in the past. This study shows that clinicians should consider vector-borne diseases as potential differential diagnoses in cats that originate from or have travelled to endemic regions. In general, the occurrence of VBPs is associated with the distribution of their vectors, which are influenced by factors such as climate, land use and human density. Spain has a highly variable climate, ranging from a continental humid climate to a Mediterranean climate. In Zaragoza, the climate is Mediterranean, with a marked continental influence, characterized by low rainfall (320 mm per year) and moderate average temperatures (15.3 °C). However, the confluence in the study area of several rivers may favour the existence of differentiated environments. This particularity could explain the results obtained in the central area (Table 3), with a higher number of animals testing positive for the pathogens evaluated in this study.

Seasonal distribution was also been analysed in the present study. We detected seasonal differences mainly in *Hepatozoon* spp., haemotropic *Mycoplasma* spp. and *L. infantum*. For *Hepatozoon* spp., more positive samples were detected in spring, followed by autumn and finally summer. Although little is known about the epidemiology of *Hepatozoon* in cats, arthropod activity in these seasons in Spain could pose a risk to free-roaming cats. For haemotropic *Mycoplasma* spp., a higher number of positive animals were detected in the winter and a possible justification could be that during this season the animals are more likely to live in groups, favouring the transmission of these pathogens. Finally, *L. infantum* has been detected more frequently in animals in winter, which might be explained by the variable and long incubation time after sandfly infection during the warmer seasons.

We observed differences between the regional distributions of selected VBPs in Zaragoza (Table 3). The comparison of results between this study and other studies published in Spain and other European Mediterranean countries is difficult and not always possible. The highly statistically significant impact of rainfall on *Hepatozoon* spp. with a strong positive correlation (*P* = 0.005, *P* = 0.753) is remarkable. To the authors’ knowledge, this type of association has never published before.

Co-infections were recognized in 35.4% (56/158) of the cats that tested positive. One cat (1.8%) had a quadruple infection, 12 cats (21.4%) had a triple infection, and 43 cats (76.8%) had a double infection. Co-infections with multiple VBPs can complicate diagnosis and treatment in dogs. A higher percentage of co-infections with other VBPs may lead to more marked laboratory abnormalities and severity in the case of canine leishmaniosis [25]. However, limited information is available in cats, although it is plausible that co-infections could predispose cats to immune system exhaustion.

*Hepatozoon* sp. DNA was detected in 25.6% (85/332) of the stray cats in this study. The diagnosis of *Hepatozoon* spp. in cats from Zaragoza reported herein supports that this protozoan is widespread on the Iberian Peninsula, considering the descriptions recently reported in cat populations from Portugal and different regions of Spain [7, 26–29]. The *Hepatozoon* infection prevalence described in previous Spanish studies varied from 0.6% [28] to 4% in cats from the Barcelona area [29] and was as high as 16% in a cat colony from Barcelona [7], in all cases lower than the prevalence reported in our study. These differences in the number of cats that tested positive could be due to the vector distribution, characteristics of the cat populations and differences in the molecular detection methods used. In the present study, the stray status of cats and possible major exposure to ectoparasites may explain the higher prevalence detected. *Hepatozoon* spp. are transmitted by ingestion of the final host containing mature oocysts by the intermediate host [30]. Although the vectors of feline hepatozoonosis are still unknown, it is expected that *H*. *felis* is transmitted by a haematophagous arthropod, as demonstrated for other *Hepatozoon* spp. transmitted by fleas, ticks, mites, lice, mosquitoes and sand flies [11]. Variations in the possible vector distribution can explain, at least partially, the differences in *Hepatozoon* sp. infection rates described in studies performed in different regions. Other transmission modes have been described for some *Hepatozoon* spp., including intrauterine transmission and carnivorism [12, 31–33].

*Hepatozoon canis* was detected in one of the cats (catalogue #98). Although *H. canis* is usually found in dogs and wild carnivores, it was previously also detected in cats from other countries, including France, Italy and Israel [12, 13, 34].

In the present study, a high haemotropic *Mycoplasma* sp. infection percentage (107/332, 32.2%) was recorded, with *Candidatus* M. haemominutum most frequently detected (61/332, 18.4%), followed by *M. haemofelis* (35/332, 10.5%) and *Candidatus* M. turicensis (11/332, 3.1%). These data are quite similar to those from previous studies in Spain. In the Barcelona region, the previously reported haemotropic *Mycoplasma* sp. prevalence ranged from 7.8% to 11.9% [35, 36], and it was 10.6% in Madrid [37]. In Italy, similar prevalence estimates (11.6% to 18.3%) were reported [1, 3, 38, 39]. However, higher haemotropic *Mycoplasma* spp. prevalence has been reported in Cyprus (26.4%) [40] and Portugal (27.1%) [41]. Haemotropic *Mycoplasma* sp. are frequent in European cats, with slight differences within the countries. A higher risk of testing positive for haemotropic *Mycoplasma* spp. in male cats than in female cats was also demonstrated in a study of cats in Bangkok, Thailand [42], which is consistent with our results.

The occurrence of *Bartonella* sp. in cats from different areas of Spain is reported with frequencies ranging from 0.3 to 38.3% [29, 35, 43–45]. The detection of *Bartonella* sp. infection (9.3%) in stray cats from Zaragoza reported herein confirms that these bacteria are widespread in Spain, similar to previous observations in cats from other areas (11.9% in Barcelona) of the country [36]. Furthermore, our results are in line with those of previous studies investigating free-roaming cats in Germany (16.3%) [46] and Italy (18%) [47]. In contrast, the *Bartonella* sp. prevalence estimate reported herein is higher than that recorded in studies investigating pet cats with indoor lifestyles in the USA (< 5.0%) [48] or Egypt (3.0%) [49], highlighting the different risk of cats being exposed to risk factors (e.g. ectoparasite infestation) based on their lifestyle.

Based on the heterogeneity of the ITS and 16SrRNA region sequences, the species *B. henselae* includes genotype I (Houston I) and genotype II (Marseille) and different subtypes [24, 50]. The two genotypes display geographical heterogeneity, wherein genotype II is prevalent in European countries and the USA [1, 3, 46, 51], while genotype I is mainly reported in Asia and North Africa [49, 52, 53]. In the present study, *B. henselae* was the unique species detected, with 16S rRNA/ITS type II (8.4%) being found more frequently than type I (0.3%), in line with what has already been documented in Western countries.

The cat is the main animal host reservoir of *Bartonella henselae*, *B. clarridgeiae* and *B*. *koehlerae* [18, 54]. *Bartonella clarridgeiae* and *B*. *koehlerae* were not detected in stray cats from the Zaragoza region in the present study, although their presence cannot be confidently excluded in the area, as two of the samples that tested positive by generic qPCR could not be re-amplified by conventional PCR because of the low DNA quantity available, so the identity of these two samples could not be determined.

Of interest, *Bartonella* sp. and haemotropic *Mycoplasma* spp. co-infection (7/332, 2.1%) was observed herein, similar to in previous studies conducted in the Barcelona region (4.4%) [36] and in Italy (0.1% to 3%) [1, 38, 39].

In the present study, the *Leishmania* sp. infection percentage was 2.1% (7/332). In Spain, PCR positive percentages of 5.6% and 6% were recorded in blood samples from cats in Zaragoza [55] and Murcia [56], respectively. A study performed by Ortuno et al. (2023) revealed a higher detection percentage of the pathogen by PCR in the skin and lymphoid tissue than in the blood of cats and in the skin of healthy cats than in the skin of cats with clinical signs [56]. Another study suggested that PCR testing of conjunctival swabs is more sensitive test than that of peripheral blood [57]; however, this is highly dependent on the cellularity of the swabs. The relatively low infection percentage observed in the present study may thus in part be explained by the type of sample used, as demonstrated for canine leishmaniosis as well [58], and the fact that only clinically healthy stray cats were included in the study.

*Anaplasma phagocytophilum, A. platys* and *E. canis* DNA was not detected in this study. The prevalence of these two tick-transmitted genera described in previous Spanish studies varied from 0 to 1% [36]. In contrast, a high molecular prevalence of *E. canis* (9.9%) and *A. phagocytophilum* (8.4%) was described in cats from Madrid, Spain [43]. *Anaplasma* and *Ehrlichia* infections in cats are generally rare in southern European countries [36, 38, 59], which is in accordance with the results of the present study. For *A. phagocytophilum*, this is probably due to the distribution of ticks of the *Ixodes persulcatus* complex as competent vectors, which are only rarely detected in Mediterranean countries and more frequently found in Central or Northern Europe [60].

Rickettsia spp., Piroplasmida (*Babesia* spp., *Theileria* spp., *Cytauxzoon* sp.) and microfilariae (*D*. *immitis*, *D. repens*, *Acanthocheilonema reconditum*) DNA was not detected in the present study. Feline piroplasmid infection is quite common in Europe. Piroplasmid infection is caused by *Cytauxzoon* sp., with a prevalence in Spain of 1.2% [8]. In Italy, a few reports of *Cytauxzoon* sp. infection have been published from the northern and central regions [61, 62]. Other piroplasms usually associated with dogs, such as *Babesia vogeli* [63] and *Babesia canis* [27], have also been described in European cats.

Microfilariae infections in cats appear to be rare and have only been sporadically documented in cats from Mediterranean regions, although a seropositivity of 24.4% was recently reported in stray cats from Zaragoza [64], and infections of two cats by *D*. *immitis* and *D*. *repens* were recently reported in Italy [65]. However, limitations in cats associated with PCR testing should be considered, as cats most often do not show prominent numbers of microfilariae in the peripheral blood, resulting in false-negative PCR results [66].

## Limitations of this study

The limitations of this study are mainly its retrospective design, predominantly due to missing anamnesis and information regarding clinical signs consistent with vector-borne infections. Probably, no ectoparasite prophylaxis was applied in the cats included in the study, but this could not be ruled out with certainty. We were not able to include the haematological and biochemistry results of the tested cats.

## Conclusions

Almost half of the cats (47.6%) tested positive for at least one vector-borne pathogen. The cats in the present study were apparently healthy, underlining the need for epidemiological studies of stray cats in Europe to identify zoonotic and non-zoonotic pathogens and the relevance of screening domestic cats for VBPs, especially in endemic areas. Immunosuppression (FeLV-/FIV infections) and male sex may contribute to positive results in molecular tests for vector-borne infectious agents.

### Supplementary Information

**Additional file 1: Fig. S1.** Maximum likelihood (ML) phylogenetic tree genepercentaged using the RAxML plugin in Geneious 10.1.3 software, calculated from the partial internal transcribed spacer (ITS) sequences of *Bartonella* species. Sequence data genepercentaged in the present study are highlighted in bold. *Brucella melitensis* was used as outgroup. Bootstrap values > 50% are shown at the nodes.

**Additional file 2: Fig. S2.** Maximum likelihood (ML) phylogenetic tree genepercentaged using the RAxML plugin in Geneious 10.1.3 software, calculated from the partial 18S rRNA gene sequences of selected *Hepatozoon* species. Sequence data genepercentaged in the present study are highlighted in bold. *Adelina dimidiata* was used as outgroup. Bootstrap values > 50% are shown at the nodes.

**Additional file 3: Fig. S3:** Average amount of rainfall sorted by months in the time frame of the study (blue line) and historically (red line) in Zaragoza (Spain)

**Additional file 4: Fig. S4**: Average temperature sorted by months in the time frame of the study (blue line) and historically (red line) in Zaragoza (Spain).

## Data Availability

All data generated or analyzed during this study are included in this published article.

## Abbreviations

DNA: Deoxyribonucleic acid
EDTA: Ethylenediaminetetraacetic acid
FeLV: Feline leukaemia virus
FIV: Feline immunodeficiency virus
OR: Odds ratio
PCR: Polymerase chain reaction
rRNA: Ribosomal ribonucleic acid
VBP: Vector-borne pathogen
