# Supplementary figures and images for: Molecular survey on vector-borne pathogens in clinically healthy stray cats in Zaragoza (Spain)
Source: Parasit Vectors. 2023 Nov 20;16:428. doi: 10.1186/s13071-023-06046-y (PMC10662132; doi:10.1186/s13071-023-06046-y)

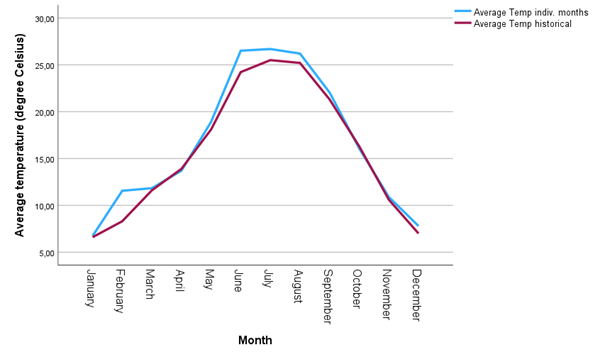

Supplement: Supplementary file 1 — Additional file 1: Fig. S1. Maximum likelihood (ML) phylogenetic tree genepercentaged using the RAxML plugin in Geneious 10.1.3 software, calculated from the partial internal transcribed spacer (ITS) sequences of Bartonella species. Sequence data genepercentaged in the present study are highlighted in bold. Brucella melitensis was used as outgroup. Bootstrap values > 50% are shown at the nodes. [file 13071_2023_6046_MOESM1_ESM.png]

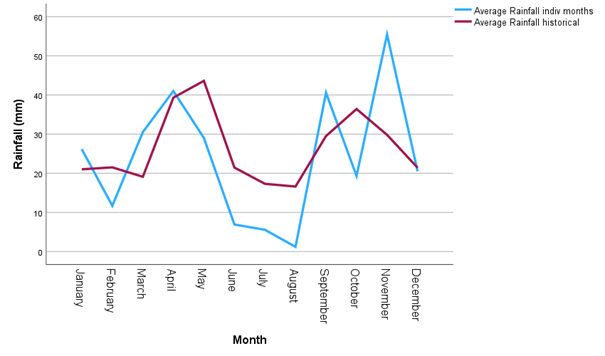

Supplement: Supplementary file 2 — Additional file 2: Fig. S2. Maximum likelihood (ML) phylogenetic tree genepercentaged using the RAxML plugin in Geneious 10.1.3 software, calculated from the partial 18S rRNA gene sequences of selected Hepatozoon species. Sequence data genepercentaged in the present study are highlighted in bold. Adelina dimidiata was used as outgroup. Bootstrap values > 50% are shown at the nodes. [file 13071_2023_6046_MOESM2_ESM.png]

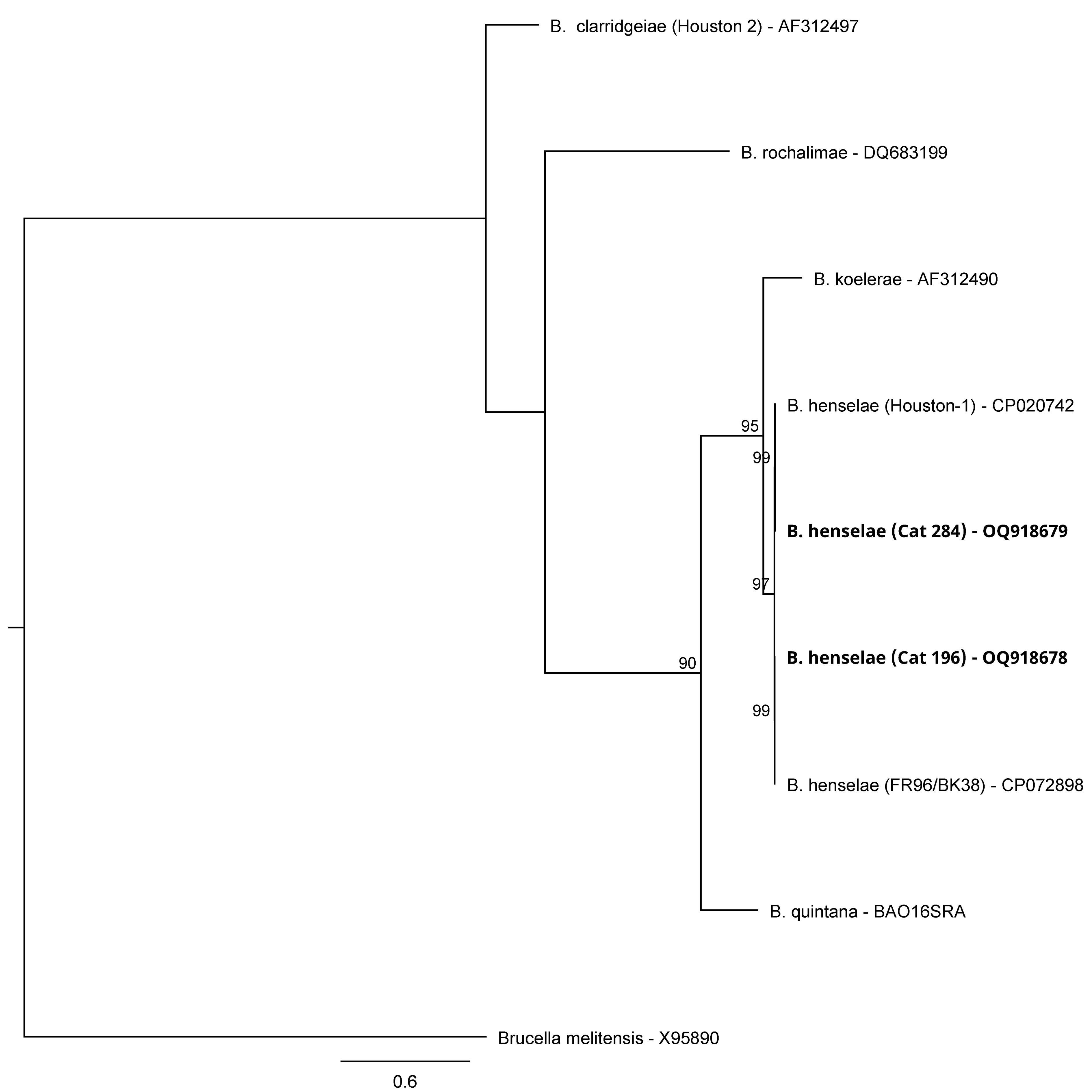

Supplement: Supplementary file 3 — Additional file 3: Fig. S3: Average amount of rainfall sorted by months in the time frame of the study (blue line) and historically (red line) in Zaragoza (Spain) [file 13071_2023_6046_MOESM3_ESM.tif]

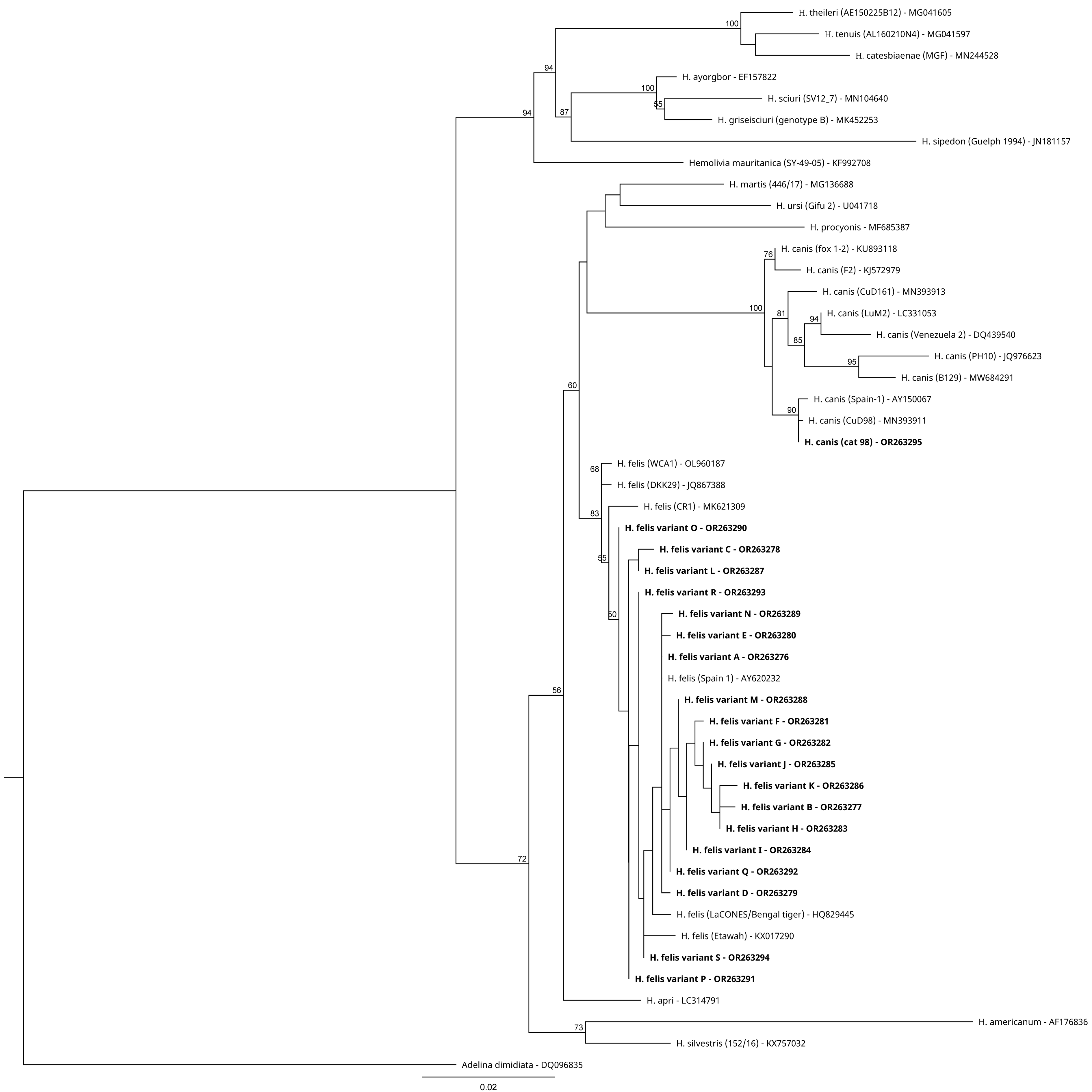

Supplement: Supplementary file 4 — Additional file 4: Fig. S4: Average temperature sorted by months in the time frame of the study (blue line) and historically (red line) in Zaragoza (Spain). [file 13071_2023_6046_MOESM4_ESM.tif]
